# The Effect of 4′-hydroxy-3,4,5-trimetoxystilbene, the Metabolite of Resveratrol Analogue DMU-212, on Growth, Cell Cycle and Apoptosis in DLD-1 and LOVO Colon Cancer Cell Lines

**DOI:** 10.3390/nu12051327

**Published:** 2020-05-07

**Authors:** Malgorzata Jozkowiak, Paulina Skupin-Mrugalska, Andrzej Nowicki, Sylwia Borys-Wojcik, Marcin Wierzchowski, Mariusz Kaczmarek, Piotr Ramlau, Jadwiga Jodynis-Liebert, Hanna Piotrowska-Kempisty

**Affiliations:** 1 Department of Toxicology, Poznan University of Medical Sciences; Dojazd 30 St., PL-60-631 Poznan, Poland; malgorzata.jozkowiak@gmail.com (M.J.); andrzej.m.nowicki@gmail.com (A.N.); pioramlau@gmail.com (P.R.); liebert@ump.edu.pl (J.J.-L.); 2 Department of Inorganic and Analytical Chemistry, Poznan University of Medical Sciences, Grunwaldzka 6, 60-780 Poznan, Poland; psmrugalska@ump.edu.pl; 3 Department of Anatomy, Poznan University of Medical Sciences, Swiecickiego 6 St., PL-60-781 Poznan, Poland; sylwiaborys@outlook.com; 4 Department of Chemical Technology of Drugs, Poznan University of Medical Sciences, Grunwaldzka 6 St., PL-60-780 Poznan, Poland; mwierzch@ump.edu.pl; 5 Department of Cancer Immunology, Chair of Medical Biotechnology, Poznan University of Medical Sciences, Garbary 15 St., PL-61-866 Poznan, Poland; markacz@ump.edu.pl; 6 Gene Therapy Unit, Department of Cancer Diagnostics and Immunology, Greater Poland Cancer Centre, Garbary 15 St., PL-61-866 Poznan, Poland

**Keywords:** resveratrol analogue, DMU-281, receptor- and mitochondria-mediated apoptosis, colon cancer

## Abstract

Resveratrol is a phytoalexin that naturally occurs in grapes, blueberries, cranberries, peanuts and many other plants. Although resveratrol inhibits carcinogenesis in all three stages, its clinical application is restricted due to poor pharmacokinetics. The methylated analogues of resveratrol have been found to have higher bioavailability and cytotoxic activity than that of the prototupe compound. Among the various methoxy derivatives of resveratrol, 3,4,5,4′-tetrametoxystilbene (DMU-212) is suggested to be one of the strongest activators of cytotoxicity and apoptosis. DMU-212 has been shown to exert anti-tumor activity in DLD-1 and LOVO colon cancer cells. Since colorectal cancer is the third most common cause of cancer-related deaths worldwide, the development of new anticancer agents is nowadays of high significance. The aim of the present study was to assess the anticancer activity of 4′-hydroxy-3,4,5-trimetoxystilbene (DMU-281), the metabolite of DMU-212, in DLD-1 and LOVO cell lines. We showed for the first time the cytotoxic activity of DMU-281 triggered via cell cycle arrest at G2/M phase and apoptosis induction accompanied by the activation of caspases-9, -8, -3/7. Furthermore, DMU-281 has been found to change the expression pattern of genes and proteins related to intrinsic as well as extrinsic apoptosis. Since the activation of these pathways of apoptosis is still the most desired strategy in anticancer research, DMU-281 seems to provide a promising approach to the treatment of colon cancer.

## 1. Introduction

According to the WHO, cancer is the most common cause of death globally and led to ~9.6 million deaths in 2018. About one in six deaths have been estimated to occur due to cancer itself. Colorectal cancer is the third major cause of cancer-related deaths worldwide (862,000 deaths in 2018) [1]. The high mortality of this tumor is mainly due to the late diagnosis at stages III and IV and impaired clinical responses to chemotherapy due to emerging drug resistance [2]. Therefore, the development of new anti-cancer agents is nowadays of high significance.

One of the most desired types of cancer cell death triggered by chemotherapeutics is apoptosis since it has been shown not to induce any inflammation in the aftermath of cancer treatment [3,4]. Apoptosis is the best known form of programmed cell death, which is mediated via two major signaling pathways, the mitochondria- and the receptor-mediated ones [5]. Many therapeutics used routinely in colorectal cancer treatment have been shown to induce both extra- and intracellular modes of apoptosis [5,6].

Resveratrol is a phytoalexin naturally found in grapes, blueberries, cranberries, peanuts and many other plants. Resveratrol displays pleiotropic biological activities, including antioxidative, anti-inflammatory, chemopreventive and anti-tumor ones [7,8]. Although resveratrol inhibits carcinogenesis at the stage of initiation, promotion and progression, its clinical application is restricted due to poor pharmacokinetic parameters.

The methylated derivatives of resveratrol have been revealed to show higher bioavailability than that of the parent compound. Since the methoxystilbenes have increased lipophilicity, they are easily transported through biological membranes into cells. Furthermore, the methylation of resveratrol has been shown to decrease the biotransformation of newly generated analogues which therefore are more resistant to degradation [9]. The structural-biological activity studies have revealed that methylated resveratrol analogues exert similar or even higher anti-proliferative activity as compared to the prototype compound. The placement of methoxy groups at positions 3,5- and 3,4,5- of the stilbene backbone has been found to play an important role in enhancing cytotoxic and pro-apoptotic effects of the compound [10].

The methylated derivative of resveratrol, DMU-212 (3,4,5,4′-tetrametoxystilbene) has been revealed to activate apoptosis in many types of human cancer cell lines, e.g., ovarian, breast, prostate, liver and colorectal ones [7,11,12,13]. DMU-212 undergoes metabolic oxidation, hydroxylation and O-demethylation reactions, as a result of which the four metabolites are generated: 3′-hydroxy-3,4,5,4′-tetrametoxystilbene (DMU-214), 4-hydroxy-3,5,4′-trimetoxystilbene (DMU-291), 3-hydroxy-4,5,4′-trimetoxystilbene (DMU-807) and 4′-hydroxy-3,4,5-trimetoxystilbene (DMU-281) [13].

DMU-281 has been revealed to exert very weak anti-proliferative activity in breast and liver cancer cell lines [14]. Concomitantly, we observed high cytotoxic effects of this metabolite in DLD-1 and LOVO colon cancer cells. Hence, the aim of the present study was to elucidate the mechanism of the anticancer effects of DMU-281 in DLD-1 and LOVO cell lines. We examined the effect of DMU-281 on apoptosis induction: the cell cycle progression, the expression pattern of apoptosis-related genes and proteins as well as the activation of caspases.

## 2. Materials and Methods

### 2.1. Chemicals and Reagents

Firstly, 3′-hydroxy-3,4,5,4′-tetrametoxystilbene (DMU-214), 4-hydroxy-3,5,4′-trimetoxystilbene (DMU-291), 3-hydroxy-4,5,4′-trimetoxystilbene (DMU-807), and 4′-hydroxy-3,4,5-trimetoxystilbene (DMU-281) were synthesized as described elsewhere [14]. The identity and purity of the compounds were established using NMR and LC–MS. The Cell Death Detection ELISAPLUS kit and the RealTime ready Human Apoptosis Panel 96 were obtained from Roche Diagnostics (Mannheim, Germany). The Caspase-Glo 3/7, Caspase-Glo 8 and Caspase-Glo 9 Assay kits were obtained from Promega Co. (San Diego, CA, USA). The Proteome Profiler Human Apoptosis Array kit was purchased from R&D Systems (Minneapolis, MN, USA). All other materials were from Sigma–Aldrich (St. Louis, MO, USA) unless otherwise stated.

### 2.2. Cell Culture and the Cytotoxicity of DMU-281 Analysis

DLD-1, LOVO and CaCo-2 colon cancer cell lines were purchased from the European Type Culture Collection. The cells were cultivated in an incubator under optimal culture conditions (temperature: 37 °C, humidity: 95%, carbon dioxide content: 5%). These cell lines were cultured in phenol red-free Dulbecco’s modified eagle medium (DMEM) complemented with 10% fetal bovine serum (FBS), 2.5 mM L-glutamine, streptomycin (0.1 mg/mL) and penicillin (100 U/mL). To assess the cytotoxic activity of DMU-281, the DLD-1, LOVO and the CaCo-2 cells were detached with a trypsin-EDTA solution and plated in 96-well plates at a density of 2 × 10^4^ cells/well in 150 µL of DMEM. After 24 h, DMU-281 was added from the stock solution prepared in DMSO at a concentration of 100 mM/mL. The final concentration of DMSO in the cell treatment mixtures was less than 0.1%. The control cells were maintained under the same conditions with 0.1% DMSO. After 24, 48 and 72 h incubation, the solution was removed and the MTT/DMEM mixture (1:8) was added. The resulting formazan crystals were then dissolved in 200 µL of DSMO. Absorbance was measured using an Elx-800 plate reader (BioTek, Winooski, VT, USA) at 570 nm (reference wavelength 650 nm). The dose of DMU-281 that was required for 50% cell growth inhibition (IC_50_) was assessed from a plot of percent cell viability versus the logarithm of concentration.

### 2.3. Apoptosis and Necrosis Assays

To assess the effects of DMU-281 on apoptosis and necrosis induction in LOVO and DLD-1 cell lines, we used the Cell Death Detection ELISA ^PLUS^ kit in accordance with the manufacturer’s instructions. Briefly, the cell suspensions were added to 96-well plates (2 × 10^4^ cells/well in 150 µL of growth medium) and treated the following day with DMU-281 at concentrations of 10 µM and 20 µM. After 72 h, the supernatant as well as the lysate were transferred to a streptavidin-coated plate and then incubated with the solution of incubation buffer, anti-histone-biotin and anti-DNA-peroxidase. Two hours later, the unbound antibodies were removed by washing with phosphate-buffered saline (PBS). Quantification of the nucleosomes was achieved by measuring the optical absorbance at 405 nm (reference wavelength 490 nm) against the substrate solution as a blank.

### 2.4. Cell Cycle Analysis

The cell cycle was assessed with the use of propidium iodide as follows. When the cultures were finished, the cells were fixed with 70% ethanol and kept for twenty-four hours at −80 °C. Then, the cells suspended in PBS were centrifuged at 1200 rpm at 4 °C for 5 min. Cell pellets were resuspended in propidium iodide at concentration 10 μg/mL and incubated at 4 °C. After 30 min, the stained cells were studied used the FACSCanto flow cytometer (Becton Dickinson, Franklin Lakes, NJ, USA). The acquired data were evaluated using the FACS Diva software (Becton Dickinson).

### 2.5. Assessment of Caspase-3/7, -9 and Caspase-8 Activation

DLD-1 and LOVO cells at a density of 2 × 10^4^ cells/well in 150 µL of DMEM were plated in 96-well plates and the following day DMU-281 at the concentrations of 10 µM and 20 µM was added. After 72 h, the medium was removed and then caspase-3/7, caspase-9 and caspase-8 activation was assessed with the luminescent Caspase-Glo^®^-3/7, -9 and -8, assay kits (Promega, San Diego, CA, USA) following the producer’s instructions. Luminescence was measured using the Tecan i-control (Mannedorf, Switzerland).

### 2.6. PCR Array

After 72 h incubation with DMU-281 (20 µM), the DLD-1 and LOVO cells were harvested and the RNA was extracted according to Chomczynski and Sacchi [15]. The integrity of RNA was established using agarose gel electrophoresis. The concentration of RNA was assessed by the measuring of the absorbance at 260 nm. The RNA samples were exposed to DNase I enzyme and reverse transcribed into cDNA using oligo-dT primers.

PCR array analysis was performed in a LightCycler Instrument 480 (Roche Diagnostics, Mannheim, Germany) using a LightCycler 480 Probes Master kit. The relative quantification was used to determine target cDNA. cDNA was not added to the negative control samples.

To conduct PCR array assays, the RealTime ready Human Apoptosis Panel 96 (Roche, Germany) was applied. For amplification, 10 µL of the reaction mixture (0.2 mL of cDNA, 4.8 mL of LightCycler 480 Probes Master and 5 mL of water) was transferred to each well of 96-well plates, in which the specific primers and probes were based. The quantity of 84 apoptosis-related genes was normalized by seven housekeeping genes.

### 2.7. Protein Expression Analysis

To assess the relative expression of the human apoptosis-related proteins level in the DLD-1 and LOVO cell lines treated with 20 µM DMU-281, the Proteome Profiler Human Apoptosis Array kit (R&D Systems) was used following the producer’s protocol. Briefly, the proteins isolated from the DLD-1 and LOVO cells were exposed to a vehicle (DMSO) or DMU-281, and were incubated overnight with the membrane-based sandwich apoptosis array at 4 °C. After the washing step, a mixture of apoptosis-detection HRP-conjugated antibodies was applied to identify the pro- and anti-apoptotic proteins by chemiluminescence. The obtained images were scanned and analyzed using the ImageJ program.

### 2.8. Statistical Analysis

Data were shown as the means ± SD for the three independent experiments. Statistical studies were carried out using one-way analysis of variance (ANOVA) and the Student–Newman–Keuls test. The GraphPad Prism 6.0 version for Windows was used for this purpose. *p* values less than 0.05, 0.01 and 0.001 were taken as statistically significant.

## 3. Results

### 3.1. Effect of the Metabolites of DMU-212 on Cells Viability and Cell Cycle

To investigate the cytotoxic effects of the four metabolites in DLD-1, LOVO and CaCo-2 colon cancer cells, an MTT assay was performed after 24 h, 48 h and 72 h at concentration ranges of 0–20 µM. The viability of the DLD-1, LOVO and the CaCo-2 cells treated with the highest concentration 20 µM of DMU-281 for 72 h was reduced to 44.87% ± 7.57%, 50.71% ± 14.94% and 84.87% ± 11.56%, respectively (Table 1).

Based on the results presented in Table 1, DMU-281 was chosen for further investigation, and Caco-2 cells have been excluded due to their resistance to this compound.

**Figure 1:**
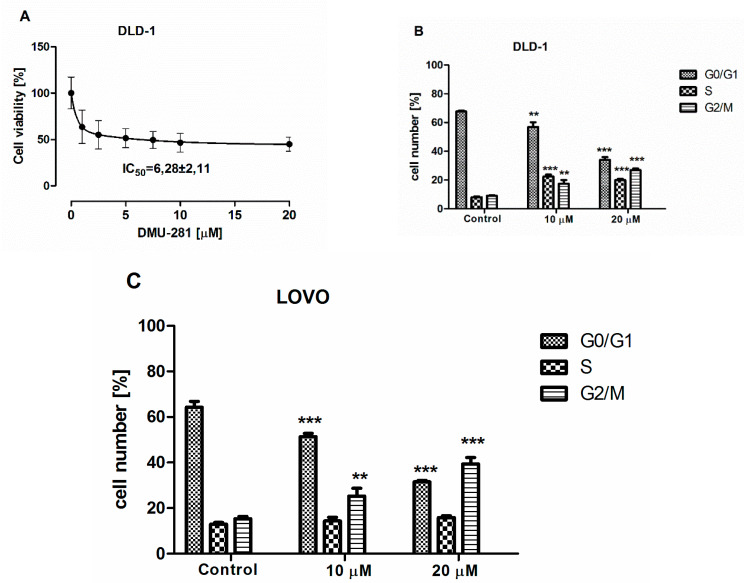
Effect of DMU-281 on the cell viability and cell cycle. (**A**) The 50% cell growth inhibition (IC_50_) value was assayed by the MTT test in the DLD-1 cells exposed to DMU-281 for 72 h at the concentration range of 0–20 µM. Cell cycle analysis was obtained by flow cytometry in (**B**) DLD-1 and (**C**) LOVO cells treated for 72 h with a vehicle or with 10 µM and 20 µM of DMU-281. Results of the three independent replicates are presented as the mean ± SD. *** *p* < 0.001 and ** *p* < 0.01.

DMU-281 was the only one metabolite of DMU-212 that reduced the viability of DLD-1 cells by 50% at a concentration of 6.28 ± 2.11 µM after 72 h of incubation (Figure 1A). Flow cytometry was conducted to perform cell cycle analysis. A significant increase in the number of the DLD-1 cells exposed to 10 µM and 20 µM was found at G2/M phase by 1.5- and ~2.5-fold decrease when compared to the controls (Figure 1B). As shown in Figure 1C, LOVO colon cancer cells were arrested with 10 µM and 20 µM of the compound tested in the G2/M phase by ~80% and ~170%, respectively. The DLD-1 and LOVO cells treatment with the DMU-281 at both concentrations also evoked a statistically significant decrease in the number of cells in the G0/G1 phase when compared to the controls (Figure 1B,C).

### 3.2. Apoptosis Induction by DMU-281

**Figure 2:**
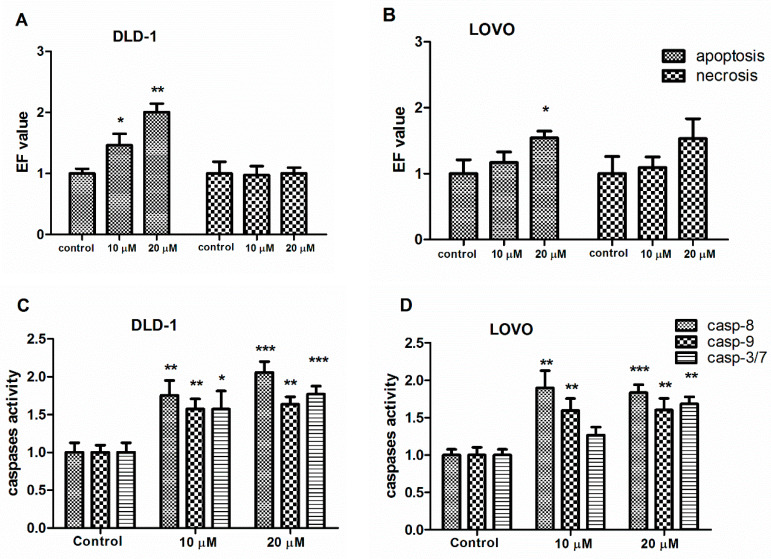
Apoptosis induction by DMU-281. DLD-1 and LOVO cells were exposed to a vehicle or 10 µM and 20 µM of DMU-281 for 72 h. Apoptosis and necrosis induction was determined by the ELISA assay and expressed as an enrichment factor (EF) in the DLD-1 (**A**) and LOVO (**B**) cells. Caspase-8 (Casp-8), caspase-9 (Casp-9) and caspase-3/7 (Casp-3/7) activities were assayed in the DLD-1 (**C**) and the LOVO (**D**) cell lines. Results of the three independent replicates are presented as the mean ± SD. *** *p* < 0.001, ** *p* < 0.01 and * *p* < 0.05.

The activation of apoptosis in the DLD-1 and LOVO colon cancer cells treated with 10 µM and 20 µM of DMU-281 for 72 h was analyzed by the Cell Death Detection ELISA ^plus^ test. The pro-apoptotic effect of DMU-281 was shown as an enrichment factor (EF), (Figure 2A,B).

Both concentrations of DMU-281 (10 µM and 20 µM) caused a statistically significant up-regulation of the level of nucleosomes in the DLD-1 lysate compared to the untreated cells, EF = 1.46 and EF = 2.01, respectively (Figure 2A). The statistically substantial pro-apoptotic activity of DMU-281 was also observed in the LOVO cells, however this was only after exposure to the highest concentration tested (20 µM) and the induction of apoptosis was less pronounced than in DLD-1 cells, EF= 1.54 (Figure 2B). The number of necrotic cells in the DLD-1 and LOVO supernatants was also analyzed; no statistically significant differences as compared to control were noted (Figure 2A,B).

The activation of caspase-3/7 was determined by the Caspase-Glo 3/7 test. The statistically significant up-regulation of the activity of effector caspase-3/7 occurred in the DLD-1 colon cancer cell line by ~50% and 65% following DMU-281 treatment for 72 h with 10 µM and 20 µM, respectively (Figure 2C). The activation of caspase-3/7 was increased by 60% in LOVO cells exposed only to the higher concentration of the compound tested (20 µM). No statistically significant effect of DMU-281 at a concentration of 10 µM was found (Figure 2D). Figure 2C,D shows that the activations of initiator caspases-8 and -9 were up-regulated in the DLD-1 and LOVO cell lines treated with both concentrations tested (10 µM and 20 µM) compared to the untreated controls. However, the effect of DMU-281 on the caspase-8 activity was more pronounced in both the DLD-1 (↑~75 %) and the LOVO cells (↑~50 %) as compared to caspase-9.

### 3.3. The Changes in the Expression of Apoptosis-Related Genes by DMU-281

**Figure 3:**
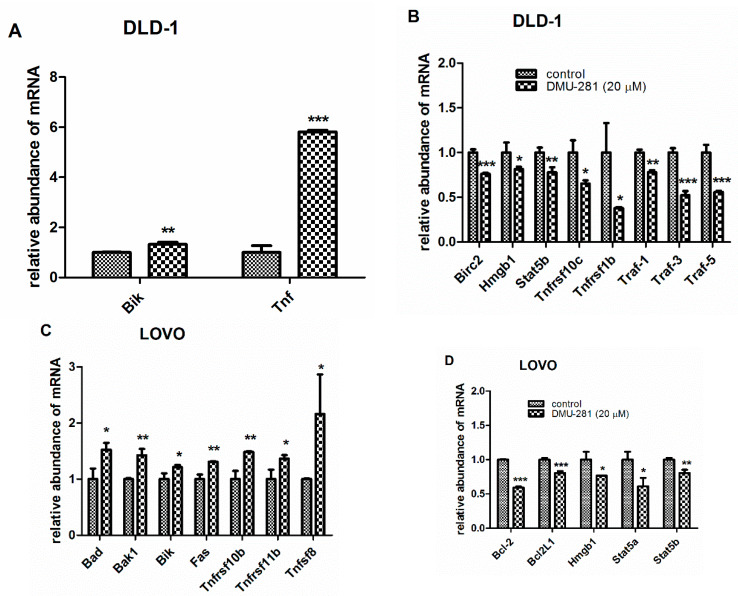
The changes in the expression of the apoptosis-related genes induced by DMU-281. The PCR array was conducted to assess the expression profiles of apoptosis-related genes in the DLD-1 (**A**,**B**) and the LOVO (**C**,**D**) cells treated for 72 h with 20 µM of DMU-281. The expression of the pro-apoptotic Bik and Tnf (**A**), and the anti-apoptotic Birc2, Hmgb1, Stat5b, Tnfrsf10c, Tnfrsf1b, Traf-1, Traf-3 and Traf-5 (**B**) genes in the DLD-1 cells. The expression changes of the pro-apoptotic Bad, Bak1, Bik, Fas, Tnfrsf10b, Tnfrsf11b and Tnfsf8 (**C**) and the anti-apoptotic Bcl-2, Bcl2L1, Hmgb1, Stat5a and Stat5b (**D**) genes in the LOVO cell line. Data are expressed as the mean ± SD (N = 3). *** *p* < 0.001, ** *p* < 0.01 and * *p* < 0.05 compared to vehicle.

To clarify the process by which DMU-281 induces apoptosis, the expression of 84 pro- and anti-apoptotic genes in DLD-1 and LOVO colon cancer cells was studied. Statistically significant changes were observed in the expression pattern of 10 apoptosis-related genes in the DLD-1 cells exposed to 20 µM of DMU-281 for 72 h (Figure 3A,B).

DMU-281 slightly induced the increase of the pro-apoptotic gene Bik while the expression of Tnf was six-fold upregulated when compared to the controls (Figure 3A). Figure 3B shows that DMU-281 triggered the suppression of the mRNA levels of five anti-apoptotic Birc2, Hmgb1, Stat5b, Tnfsrf10c, and Traf-1 genes by ~20% (Figure 3B). Concomitantly, a significant down-regulation of the expression profile of the four anti-apoptotic mRNA of Tnfsrf1b, Traf-3, and Traf-5 from 65% to 40% was found when compared to the untreated controls. In the LOVO cells treated for 72 h with DMU-281 (20 µM), we noted increased levels of the pro-apoptotic genes: Bad, Bak1, Bik, Fas, Tnfsrf10b, Tnfsrf11b and Tnfsf8 (Figure 3C), while a decreased expression by ~50%–30% of the anti-apoptotic genes: Bcl-2, Bcl2L1, Hmgb1, Stat5a, and Stat5b was found (Figure 3D).

### 3.4. The Changes in Pro- and Anti-Apoptotic Proteins Expression by DMU-281

**Figure 4:**
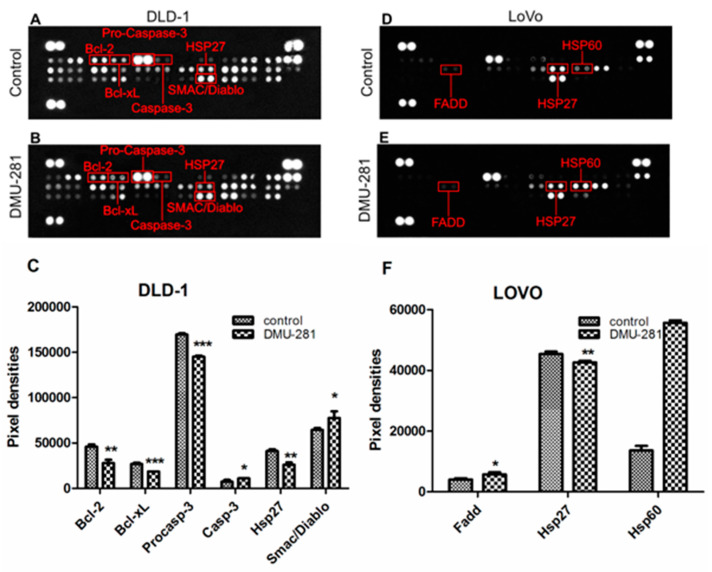
The changes in the apoptosis-related proteins expression by DMU-281. A representative picture of the apoptosis array assays. (**A**,**B**) Expression changes in the levels of Bcl-2, Bcl-xL, Procasp-3, Casp-3, Hsp27 and Smac/Diablo in the DLD-1 cells and (**D**,**E**) Fas-associated death domain protein (Fadd), Hsp27 and Hsp60 in the LOVO cells are highlighted in red in the scanned image. Densitometric studies were performed to assess the level of the indicated proteins in (**C**) the DLD-1 and (**F**) the LOVO cell lines treated with a vehicle or DMU-281 for 72 h. * *p* < 0.05, ** *p* < 0.01 and *** *p* < 0.001 compared to control.

A Human Apoptosis Proteome Profiler™Array was used to investigate the changes in the apoptosis-related proteins level induced by DMU-281 at a concentration of 20 µM in the DLD-1 and the LOVO cells after 72 h incubation (Figure 4A–F). In the DLD-1 cell line, the protein analyses revealed a statistically significant increase in the level of pro-apoptotic proteins: Casp-3 and Smac/Diablo by ~25%–30% when compared to the untreated controls (Figure 4A–C). Simultaneously, DMU-281 decreased the Procasp-3 protein expression by ~20%. The levels of the anti-apoptotic proteins Hsp27, Bcl-2 and Bcl-xL was decreased by ~40% and 30%, respectively. 

The LOVO cells treatment with DMU-281 resulted in an up-regulation of the pro-apoptotic proteins levels: Hsp60 and Fas-associated death domain protein (Fadd) by ~20% and 50%, respectively (Figure 4D–F). Concurrently, DMU-281 caused a slightly lower expression of the anti-apoptotic protein Hsp27 when compared to the untreated control.

The changes in the expression profiles of the apoptosis-related proteins and genes in the DLD-1 and the LOVO cells exposed to DMU-281 are presented in Table 2.

## 4. Discussion

In the first stage of our study, we assessed the cytotoxicity of 4′-hydroxy-3,4,5-trimetoxystilbene (DMU-281), one of the metabolites of 3,4,5,4′-tetrametoxystilbene (DMU-212), in DLD-1, LOVO and CaCo-2 colon cancer cell lines. We found that CaCo-2 cells were much less sensitive to DMU-281 compared to the DLD-1 and LOVO ones. Hence, we excluded the CaCo-2 cell line from our experimental design. The overexpression of Bcl-2 and/or Bcl-xL in tumor cells is commonly known to block the proceeding of apoptosis [16]. The CaCo-2 colon cancer cell line, in contrast to DLD-1 and LOVO, was revealed to overexpress the Bcl-2 and Bcl-xL proteins [17,18,19]. Accordingly, we can suggest that the resistance of CaCo-2 cells to DMU-281 is a result of the notably high expression of these BCL-2 family molecules.

The primary cell cultures, as more physiological models based on the genetic features of individual patient tumors, are essential tools for predicting the response to the therapy and clinical outcomes [20]. On the contrary, the established cell lines have been used for drug screening, to describe the biology of specific tumors and to identify the optimal drug candidates for therapy [21]. Since our study was the first one in which the anti-tumor activity of DMU-281 was investigated, we performed the experiments using immortalized colon cancer cell lines. Simultaneously, these cells were considered as the standard for the comparison of the anti-proliferative effects of DMU-281 with the results of our previously published data showing the anti-tumor activity of the parent compound, DMU-212 in the DLD-1 and the LOVO cell lines [22]. The higher cytotoxicity of DMU-212 was found in the DLD-1 cells rather than the LOVO ones [22]. Accordingly, in this study we observed a more potent cytotoxic effect of DMU-281 in the DLD-1 cells since they were inhibited by 50% at a concentration of 6.28 ± 2.11 µM. In the LOVO cell line, the IC_50_ value was not determined as the inhibition was 49.29% at the highest concentration tested (20 µM). However, the sensitivity of both the DLD-1 and the LOVO colon cancer cell lines to DMU-281 was lower when compared to DMU-212. The mechanism of the anti-cancer activity of DMU-212 in these cells has been assayed at concentrations 5 µM and 10 µM [22]. Since we found the lower cytotoxic potency of DMU-281 than that of the parent compound, the higher doses (10 µM and 20 µM) of DMU-281 were adopted in the present study. In various in vitro models, the naturally occurring resveratrol has also been shown to exert the anti-cancer activity mostly at concentrations ≥10 µM, which are higher than those of physiological ones [23,24]. Although pharmacological treatment is often accompanied by some side effects, the higher doses of potential chemotherapeutics are essentially more effective, and therefore preferably studied.

Several publications have shown that DMU-212 caused cell cycle arrest in the G2/M phase parallel to apoptosis induction in ovarian, breast, liver and colon cancer cells [11,12,25]. We confirmed these observations for DMU-281, which triggered the G2/M block accompanied by apoptosis in the DLD-1 and the LOVO cell lines, as evidenced by the increased EF values. No statistically significant differences in the number of the necrotic cells were shown. Hence, it could be suggested that necrosis does not contribute to the mechanism of the cytotoxic activity of DMU-281.

Apoptosis is commonly known to be initiated via two major pathways: the intrinsic (mitochondria-mediated) and the extrinsic (receptor-mediated) ones [26]. To assess the mechanism of the antitumor activity of DMU-281, we analyzed the expression pattern genes and proteins triggering intra- and extracellular apoptosis pathways. In the present study, we assessed the expression level of 84 pro- and anti-apoptotic genes and 36 apoptosis-related proteins in both the DLD-1 and the LOVO colon cancer cell lines.

The BCL-2 family members are crucial for the induction of apoptosis in a mitochondria-mediated manner. According to common knowledge, the anti-apoptotic proteins Bcl-2 and Bcl-xL inhibit apoptosis by blocking the release of cytochrome c, Smac/Diablo, and apoptosis inducing factor (Aif) from the intermembrane space of mitochondria [27]. In the DLD-1 cell line exposed to DMU-281, we found a significant decrease in Bcl-2 and Bcl-xL’s anti-apoptotic proteins level, while the pro-apoptotic Smac/Diablo protein was increased. The overexpression of Smac/Diablo has been shown to up-regulate the susceptibility of cancer cells to the activation of apoptosis [28,29,30]. Hence, we suggest that the greater vulnerability of the DLD-1 cells to the DMU-281 might be related to its ability to increase the expression of Smac/Diablo protein. In the cytosol, the activated Smac/Diablo molecule is known to antagonize the inhibitor apoptosis protein (Iap), thus allowing the activation of caspases (casp)-9 and -3 [31]. Although we did not find any changes in the expression of Iap in the DLD-1 cells (data not shown), the increase in the activity of casp-9 triggered by DMU-281 was observed. Casp-9 is commonly known to work as an initiator of the caspases cascade in the mitochondria-mediated pathway of apoptosis by cleaving and activating effector caspases, including casp-3/7 [32]. In the present study, DMU-281 was shown to increase the casp-3 protein level parallel to the down-regulation of procasp-3 in the DLD-1 cell line. Accordingly, the up-regulation of casp-3/7 activity was observed. Hence, we suggest that the Smac/Diablo protein, which is known to induce casp-9 and in consequence, casp-3/7, plays a crucial role in inducing intrinsic apoptosis by DMU-281 in the DLD-1 cell line. We also showed the up-regulated expression of the Bik gene, whose protein is implicated in the mitochondria-mediated pathway of apoptosis via the inhibition of Bcl-2 and Bcl-xL [33]. Since we observed the decrease in the expression of Bcl-2 and Bcl-xL corresponding to the up-regulation of Bik, Smac/Diablo, casp-3, casp-3/7, and casp-9, DMU-281 is suggested to trigger interactions between these molecules and induce mitochondria-mediated apoptosis in the DLD-1 cell line.

We noticed that DMU-281 also evoked the up-regulation of the expression of Tnf, which is one of the critical factors activating apoptosis in a receptor-mediated manner. Simultaneously, Tnf triggers various NfĸB-mediated cellular responses, including apoptosis as well as inflammation pathways, depending on the activation of the particular biological system [23]. The members of the Tnf family, such as the Traf molecules, activate the NfĸB factor, which prompts the expression of inflammatory or extrinsic anti-apoptotic genes [34,35]. Although we did not find any changes in the expression of NfĸB in the DLD-1 cell line, DMU-281 was demonstrated to decrease anti-apoptotic Traf1, Traf3, Traf5 and Birc2 transcripts level. Simultaneously, no changes in the pro-inflammatory NfĸB-related IL-6 and IL-1β expression were found (data not shown). On the other hand, DMU-281 was shown to decrease the level of the anti-apoptotic Tnfrsf10c and Tnfrsf1b genes that belong to the Tnf family. We also showed that DMU-281 triggered the induction of the activity of casp-8, which is a marker of extrinsic apoptosis [36]. Hence, we can suggest that DMU-281 evokes both receptor and mitochondrial pathways of apoptosis in the DLD-1 cells. DMU-212, the parent compound of DMU-281, was also shown to the trigger mitochondria- and receptor- mediated apoptosis pathways in ovarian, breast, liver and colon cancer cells [11,12,14,22,25]. These findings were consistent with our results since we found that DMU-281 exerted cytotoxic activity via intrinsic and extrinsic apoptosis in DLD-1 and LOVO colon cancer cells.

In the LOVO cell line, DMU-281 was shown to increase the pro-apoptotic Tnfrsf10b, Tnfrsf11b, Tnfsf8 and Fas genes belonging to TNF family, which is commonly known to mediate the extrinsic apoptosis pathway. Fas-associated death domain protein (Fadd) recruits casp-8 and activate executive casp-3/7 [37]. Accordingly, in the current study, DMU-281 was observed to up-regulate the expression of the Fadd protein level accompanied by the increase in the activity of casp-8 as well as casp-3/7. Hence, the extrinsic apoptosis is suggested to be involved in the mechanism of the cytotoxic effect of DMU-281 in the LOVO cells. However, we also demonstrated that DMU-281 caused an up-regulation of the mRNA levels of Bad, Bak1 and Bik parallel to a down-regulation in the Bcl-2 and Bcl-2L1 genes, whose proteins—as mentioned above—interact which each other and play an essential role in the mitochondria-mediated apoptosis pathway. Furthermore, DMU-281 was shown to downregulate the Hsp27 protein level parallel to the increase in the activity of casp-9. These results are supposed to strengthen our hypothesis of DMU-281-triggered mitochondrial apoptosis in LOVO cells, since Hsp27 is known to suppress intrinsic apoptosis via the indirect inhibition of procaspase-9, leading to the failure of caspase activation [38,39,40]. Moreover, we showed that DMU-281 caused a significant increase in the expression of the pro-apoptotic Hsp60 protein, which is also involved in the mitochondria-mediated apoptosis pathway [41].

Several studies have demonstrated the overexpression of the Stat5 protein in colorectal cancer cells. Simultaneously, Stat5a and Stat5b have been found to induce the growth of tumor cells, the progression of the cell cycle and metastasis through the deregulation of the expressions of genes related to intrinsic apoptosis [42,43]. Our findings are in line with the other authors’ results since we observed that DMU-281 caused a down-regulation of Stat5 expression in DLD-1 and LOVO cells accompanied by inhibiting the growth and cell cycle arrest. The decreased expression of the Hmgb1 mitochondrial apoptosis-related gene was shown in both the LOVO and the DLD-1 cell lines. Since the interaction between the members of the STAT family and Hmgb1 has been revealed [44,45], we can suggest that Hmgb1 is also involved in the mechanism of the cytotoxic activity of DMU-281.

## 5. Conclusions

Summing up, our study showed for the first time the cytotoxic effect of DMU-281 triggered via cell cycle arrest at the G2/M phase as well as apoptosis induction in DLD-1 and LOVO colon cancer cells. Since the activation of mitochondria- and receptor-mediated apoptosis is still the most desired strategy in anticancer research, DMU-281 might be suggested to be a potential therapeutic for colon cancer.

## Figures and Tables

**Table 1:** The viability of DLD-1, LOVO and CaCo-2 cell lines treated with metabolites of DMU-212.

Metabolites of DMU-212	Time Exposure	Cell Viability [%]		
		DLD-1	LOVO	CaCo-2
DMU-214	24 h	66.97 ± 7.14 **	82.16 ± 5.91 *	104.90 ± 2.91
	48 h	59.52 ± 12.78 **	53.60 ± 5.54 ***	80.60 ± 26.47
	72 h	56.55 ± 19.04 **	54.88 ± 12.18 ***	68.28 ± 15.84 **
**DMU-281**	24 h	89.50 ± 7.40	78.83 ± 7.93 *	90.09 ± 8.40
	48 h	62.02 ± 5.78 ***	56.37 ± 3.20 ***	90.73 ± 11.49
	**72 h**	**44.87 ± 7.57** **	**50.71 ± 14.94** ***	84.87 ± 11.56 *
DMU-291	24 h	102.99 ± 9.05	94.30 ± 6.94	107.47 ± 5.90
	48 h	94.90 ± 19.43	78.15 ± 12.44 **	97.53 ± 17.38
	72 h	75.90 ± 20.29	88.07 ± 9.09 *	85.37 ± 21.46
DMU-807	24 h	92.53 ± 8.60	79.92 ± 2.58 ***	89.63 ± 2.01 *
	48 h	95.32 ± 8.62	69.41 ± 16.59 *	77.96 ± 4.68 **
	72 h	101.18 ± 9.99	76.71 ± 25.15	80.59 ± 8.21 **

Cell lines were exposed to vehicle or compounds tested (20 µM) for 24 h, 48 h and 72 h. The results of the three independent replicates are presented as the mean ± SD. The bold numbers are dedicated to 4′-hydroxy-3,4,5-trimetoxystilbene (DMU-281) as the most cytotoxic metabolite of 3,4,5,4′-tetrametoxystilbene (DMU-212) in DLD-1 and LOVO colon cancer cell lines. *** *p* < 0.001, ** *p* < 0.01 and * *p* < 0.05 indicate a significant difference from the controls. DMU-214: 3′-hydroxy-3,4,5,4′-tetrametoxystilbene; DMU-291: 4-hydroxy-3,5,4′-trimetoxystilbene; DMU-807: 3-hydroxy-4,5,4′-trimetoxystilbene.

**Table 2:** The genes and the proteins expression patterns in the DLD-1 and the LOVO cell lines treated with DMU-281.

Cell Line	Genes Expression Profile	Proteins Expression Profile
**DLD-1**	↑ Bik, Tnf	↑ Casp-3, Smac/Diablo
	↓ Birc2, Hmgb1, Stat5b, Tnfrsf10c, Tnfrsf1b, Traf-1, Traf-3, Traf-5	↓ Bcl-2, Bcl-xL, Procasp-3, Hsp27
**LOVO**	↑ Bad, Bak1, Bik, Fas, Tnfrsf10b, Tnfrsf11b, Tnfsf 8	↑ Fadd, Hsp60
	↓ Bcl-2, Bcl-2L1, Hmgb1, Stat5a, Stat5b	↓ Hsp27

↑- increase in the expression of genes / proteins;↓- decrease in the expression of genes / proteins.
